# Advances in the Field of Micro- and Nanotechnologies Applied to Extracellular Vesicle Research: Take-Home Message from ISEV2021

**DOI:** 10.3390/mi12121563

**Published:** 2021-12-16

**Authors:** Silvia Picciolini, Francesca Rodà, Marzia Bedoni, Alice Gualerzi

**Affiliations:** IRCCS Fondazione Don Carlo Gnocchi Onlus, 20148 Milan, Italy; spicciolini@dongnocchi.it (S.P.); froda@dongnocchi.it (F.R.); mbedoni@dongnocchi.it (M.B.)

**Keywords:** extracellular vesicles, nanomedicine, nanotechnology, theranostics

## Abstract

Extracellular Vesicles (EVs) are naturally secreted nanoparticles with a plethora of functions in the human body and remarkable potential as diagnostic and therapeutic tools. Starting from their discovery, EV nanoscale dimensions have hampered and slowed new discoveries in the field, sometimes generating confusion and controversies among experts. Microtechnological and especially nanotechnological advances have sped up biomedical research dealing with EVs, but efforts are needed to further clarify doubts and knowledge gaps. In the present review, we summarize some of the most interesting data presented in the Annual Meeting of the International Society for Extracellular Vesicles (ISEV), ISEV2021, to stimulate discussion and to share knowledge with experts from all fields of research. Indeed, EV research requires a multidisciplinary knowledge exchange and effort. EVs have demonstrated their importance and significant biological role; still, further technological achievements are crucial to avoid artifacts and misleading conclusions in order to enable outstanding discoveries.

## 1. Introduction

Extracellular Vesicles (EVs) are naturally produced nanoparticles that are released by all body cells in both physiological and pathological conditions. Despite an initial underestimation of EV functions in tissue homeostasis, in the last decade, there has been a growing interest and awareness of their role in cell-to-cell communication and consequent implications in relation to multiple cellular processes and diseases [1,2,3].

Each year, the continuously growing and expanding field of EV research finds its major stage during the annual meeting of the International Society for Extracellular Vesicles (ISEV). The ISEV has been the leading society for researchers and scientists involved in the study of EVs since 2012. ISEV’s annual international meetings bring together researchers from all over the world, giving the opportunity to students and early career researchers to share their data with experienced scientists of the field. In 2021, the ISEV annual meeting (ISEV2021) was held virtually; nonetheless, it was a dynamic and active occasion for knowledge sharing and networking opportunities [4].

In the present brief review, we first summarize the main physico-chemical properties of EVs and we then report what we perceived to be the most interesting findings presented at ISEV2021 in the field of micro- and nanotechnology to inspire the *Micromachines* readership and stimulate exchange among scientists from different fields. References to proceedings published in the abstract book are reported in brackets, together with publications in extenso, where possible.

## 2. Extracellular Vesicles

**Figure 1:**
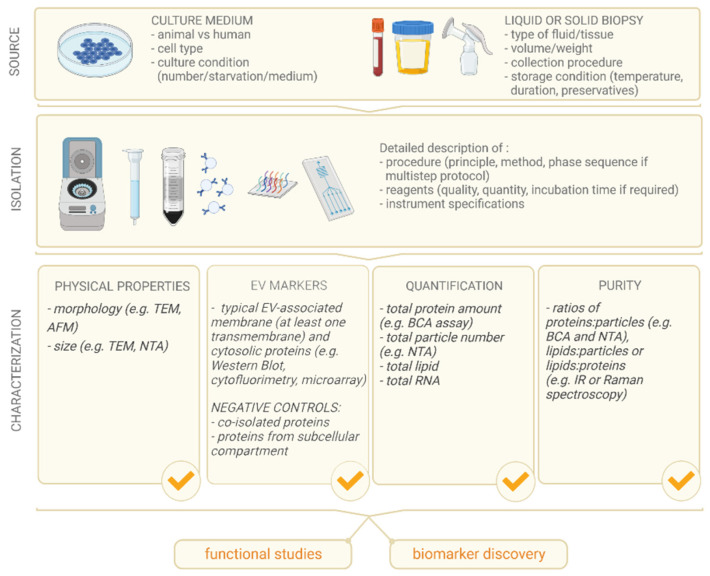
Schematic representation of the main MISEV2018 guidelines regarding the detailed description of the EV source, the isolation method used and/or developed and the general EV characterization. All these steps are crucial for (i) the reproducibility of the results, (ii) the reproducibility of downstream functional studies and (iii) the robustness of new suggested biomarkers.

EVs are bilipid membrane-enclosed nanoparticles that originate from the parental cells by means of two major pathways: (1) from the endosomal compartment by means of the fusion of multivesicular bodies (MVB) with the plasma membrane; (2) by shedding/direct budding from the plasma membrane. Besides being divided based on the mechanism of release, EVs might also be categorized by their size (medium/large and small) or density (low, middle and high), while from an antigenic point of view, they can be described based on the presence of specific membrane-bound or soluble markers [5]. The ISEV community has proposed guidelines [5,6] (Figure 1) for the proper characterization of EVs upstream to specific applications, in order to help scientists and researchers in the field to report the essential features of samples based on shared experience-based data, avoiding misleading nomenclature.

The key and most attracting feature of EVs is their intrinsic ability to mirror their parental cell status and content. Indeed, EVs were proven to express, on their membrane surface, the typical protein markers of the cell of origin, but also to maintain a biochemical signature, made up of proteins, lipids, sugars and nucleic acids, that is specific for their source cell [1,7,8,9]. Such observation has allowed researchers to develop immuno-based methods for the EV isolation and purification of specific subpopulations from culture media, tissues and liquid biopsies. Furthermore, the similarities with the cell of origin and the reported role in intercellular communication have triggered research aimed at the use of EVs in such areas as therapeutics, both in their natural state or in an artificially loaded state. As an example, EVs released by stem cells or induced pluripotent stem cells (iPS) were proven to be effective in the transfer of bioactive molecules with regenerative potential [10]. Similarly, EVs from immune cells such as microglial cells were suggested as potential therapeutics for neurological disorders when activated towards a pro-regenerative phenotype [11,12], whereas dendritic-derived EVs are a valuable example of naturally secreted EVs that can be used for the immunotherapy of cancer [13,14].

The possibility to use EVs both as nanocarriers with therapeutic functionalities, but also as vehicles of biomarkers for diagnostic and prognostic applications to personalize the therapeutic intervention, make them consistent with the definition of **theranostics** [15].

Although doubts remain about EVs’ complex structural and functional interplay with cells, as well as about their dynamics between different body fluids, their remarkable potential in clinical applications as theranostics has sped up applied research in in vitro and in vivo models. However, it is worth mentioning that the EV studies on cells and animal models have to cope with the complexity of human samples before their clinical translation. Indeed, efforts to discover valuable markers in human biofluids such as blood, urine and saliva [16,17,18] have to run in parallel with efforts to develop and standardize the methodologies for EV isolation and characterization. The study of the parameters influencing and determining the variability in the upstream and downstream procedures are still ongoing and the identification of reference protocols and variables to compare data from multiple laboratories remains a major research focus, especially when dealing with complex human biofluids such as blood. As a matter of fact, the discovery of new biomarkers and new techniques is often brought only to a proof-of-concept level, tested on small (sometimes tiny) numbers of samples [19], and when the numbers increase, the validation step is hampered by the absence of a comparative, widely approved test for proper validation and/or by the paucity of reference material for the standardization of commonly used analysis platforms [20,21].

Because of the present difficulties in the standardization of the isolation procedures and in the application of “cell-calibrated” techniques to natural nanoparticles, innovative strategies have been proposed [19,22,23,24,25] and new technology advancements are continuously emerging in the field of EV research. Below, we report on the recent updates in the field of micro- and nanotechnologies applied to EV research that were presented in May 2021 at the 10th ISEV Annual Meeting. Although far from being an exhaustive snapshot of the rapidly evolving landscape of EV research, we selected, from the abstract book of ISEV2021 [4], those references that are focused on the description of technological advances in (i) EV isolation and detection, (ii) EV characterization and biomarker discovery and (iii) EV applications for drug delivery.

## 3. Advances in EV Isolation and Detection

Issues related to isolation methodologies were approached by several researchers looking for fast, reproducible and specific methods. Indeed, methodological advances are still required to identify valuable methods for the rapid isolation of pure EV preparations that could be standardized for clinical application. For example, to allow for the purification of intact EV subpopulations, Dr. Kooijmans (University Medical Center Utrecht, Utrecht, The Netherlands) presented a universal magnetic bead-based capture-and-release method. It consists of the use of magnetic beads functionalized with CD9, CD63, CD81 or phosphatidylserine antibodies. Once isolated, each EV subpopulation was further characterized, thanks to the capture-and-release platform, making it possible to study if the composition, enzymatic activity and cellular uptake efficiency differ between EV subpopulations based on their surface molecule expression (PS 10.06). Magnetic particles were also proposed by STEMCELL Technologies (Vancouver, Canada) to isolate EV subtypes from biofluids based on user-defined surface markers. Indeed, vesicles were targeted directly with specific antibody complexes and magnetic particles; magnetically labeled EVs were separated from free EVs using a magnet showing a higher target marker expression than bulk EV isolation (PS11.13). Magnetic properties served also for the development of an on-chip magnetic immuno-extraction of small EVs from human plasma, as presented by Dr. Araya-Farias (Curie Institut, Paris, France; CC3.3). Within the framework of the European Union’s Horizon 2020 project (H2020 FET-OPEN program, INDEX project), immuno-extraction and fluidized bed technology were combined to isolate EVs, allowing for the capture and release of EVs in a single device with a capture efficiency of about 80%. The fluidized bed device, in combination with immune-extraction technology, needs to be tested to extract different EV subpopulations.

Moreover, a high-throughput method to isolate EVs from cell culture medium and human serum samples was presented, revealing higher or equivalent recovery and purity compared to ExtraPEG [26] or size-exclusion chromatography. The method, called MagPEG and based on the use of magnetic beads, can also allow for the protein profiling of EVs (Dr. Su, FSU College of Medicine, Tallahassee, FL, USA; PS10.03). To face the challenge of developing robust, versatile, scalable and economically viable processes for the isolation and recovery of pure and homogeneous EV populations, a novel approach was proposed in the MARVEL project (Evolving reversible iMmunocapture by membrAne sensing peptides: towaRds scalable extracellular VEsicLes isolation) presented by Dr. Cretich from CNR-SCITEC (Milan, Italy). It regards the use of peptides as an alternative class of ligands for EV capturing; MARVEL technology results in a portfolio of products, which includes, for example, a test for lab-scale and point-of-need tools for urinary EV enrichment and analysis, enabling the liquid biopsy of bladder cancer (PS10.11). Similarly, a novel size exclusion chromatography method, known as the SmartSEC HT kit, was presented by Dr. Hirschberg (VITO, Mol, Belgium) with the aim of identifying a standard method for EV-related biomarker detection in cerebrospinal fluid. The method allows the trapping of contaminants, overcoming the limitations of conventional SEC for a fast, easy, high-throughput EV isolation workflow working on a 96-well plate with limited sample volume (S10.07). A simplified SEC method for the high-quality isolation of EVs was presented by Dr. Cui (Jinan University, Guangzhou, China). This simple and reproducible method, based on the use of CL-6B columns, can recover 80% of EVs and is applicable to the isolation of small EVs from serum and cell culture medium; it requires two bulk elutions to acquire EVs and proteins separately (PS24.03).

Dr. Van Dorpe (Ghent University, Ghent, Belgium) presented an automated liquid handling workstation for the density-based separation of EVs from human body fluids in order to improve the reproducibility and specificity of this separation method. It was demonstrated that automation significantly diminished the presence of contaminant proteins such as lipoproteins in the plasma and uromodulin in urine (OD05.04).

A label-free EV automated purification system, called the LEAP (Label-free Extracellular-vesicle Automated Purification System) system, was also proposed by Dr. Chen (WellSIM Biomedical Technologies Inc, Berkeley, CA, USA) by implementing a novel strategy to achieve clog-free ultrafiltration of EVs (PS10.10). The system is based on alternating negative pressure combined with dual membrane harmonic oscillations to suppress the formation of fouling layers by resuspending particles into the liquid, thereby preserving the particle integrity. The approach allows for EV isolation and enrichment, improving the processing speed 15-fold and the enrichment of EVs 1000-fold.

Another novel purification strategy is the DNA-directed immobilization (DDI) of antibodies to fabricate an EV-based diagnostic assay [27]. As presented by Dr. Brambilla (CNR—SCITEC, Milan, Italy), DNA-antibody conjugates were used to generate microarrays that are able to selectively isolate specific EV subpopulations that can then be released using the enzymatic cleavage of a DNA linker mediated by DNase I. This platform showed consistency with commercial kits and allowed for the separation of intact vesicles, as confirmed by NTA, TEM and nanoFCM (OD05.02).

**Figure 2:**
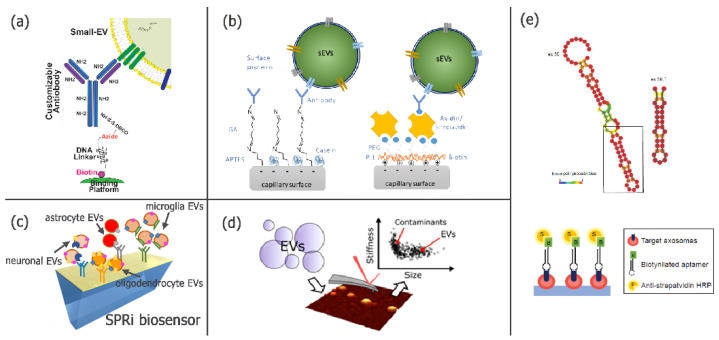
(**a**) Schematic representation of the EV-CATCHER strategy [29] developed for selectively purifying small-EVs from human biofluids. The assay relies on the binding of a degradable dsDNA-linker to a DBCO-activated antibody and to a streptavidin-coated platform. (**b**) Schematic diagrams of the APTES-GA and PPB-avidin/streptavidin functionalization method where a capillary surface is coated with a self-assembled monolayer of APTES or a layer of a PLL-PEG copolymer conjugated with biotin, which are then linked to the capture antibody. This experimental setup was used in electrokinetic measurements [30] (adapted with permission from [30]. Copyright: American Chemical Society, 2021). (**c**) Schematic representation of an SPRi-based biosensor for the isolation and simultaneous characterization of multiple EV subpopulations from liquid biopsies [31]. (**d**) Simple Atomic Force Microscopy (AFM)-based experimental procedure for the simultaneous nanomechanical and morphological analysis of EVs. The platform can be used to discriminate between subpopulations of vesicular and non-vesicular objects in the same sample [32] (adapted with permission from [32]. Copyright: American Chemical Society, 2020). (**e**) Secondary structure prediction of ex-50 aptamer and its shortened version (ex-50.T). In the ELONA-based detection system, plates are coated with EVs and incubated with biotinylated aptamers; signals are detected with streptavidin-HRP [33].

Looking for a very fast (<15 min), easy to use and economic (<1 $/each) isolation method, Dr. Jackson (Clemson University, Clemson, SC, USA) presented capillary-channeled polymer (C-CP) fiber micropipette tips employed in a solid-phase extraction workflow, which allowed processing using low sample amounts (OD05.05). The method was proven to be suitable for clinical applications in terms of sample volumes and time scales, and also to be extremely versatile as it was tested for the EV isolation from human urine, saliva, cervical mucus, serum and goat milk matrices [28]. Another purification assay was designed for the high-throughput analysis of low abundance miRNAs of EVs and the evaluation of EVs’ functional characteristics. The assay, called EV-CATCHER (EV Capture by AnTibody of CHoice and Enzymatic Release), was recently published [29] (Figure 2a), and demonstrated the possibility for use in the isolation of EVs from the serum of hospitalized patients with COVID-19 disease, with two miRNAs, associated with inflammation and endothelial cell repair inhibition, identified as being downregulated with disease severity (CC8.2).

Another efficient platform for the isolation of high pure EVs, presented by Dr. Pan (Nanfang Hospital, Guangzhou, China), is based on a defected nanoscale metal-organic frameworks (MOF) with a probe PO43-spacer-DNA-Cholesterol (PSDC) used for the binding between the phospholipid bilayer of EVs and cholesterol. The system was tested to separate EVs from plasma samples of patients with breast cancer in different clinical stages and healthy subjects, demonstrating its advantages as a fast, low cost, efficient and contaminant-free method (OD05.06).

It is worth mentioning that so many different isolation methods raise concerns about the introduction of artifacts and the eventual subgrouping of EVs. For this reason, it is crucial for authors proposing new methods to (i) accurately report the new procedure; (ii) accurately report pre-analytical variables (i.e., storage conditions, biofluid collection methods, etc.); (iii) follow the MISEV2018 guidelines of the ISEV community, in order to provide a panel of features that can be used to compare methods from multiple laboratories. In addition, efforts have been made by some researchers to compare procedures and also to evaluate their influence on downstream applications. This was the case for Dr. Phan (University of Sydney, Sydney, Australia), who presented a multiscale characterization approach to uncover the correlation between isolation methods, physico-chemical composition and EV biological functions (PS06.08). Using a range of optical and non-optical techniques, including Resonant Mass Measurement for the characterization of dry mass and buoyant mass of large EVs and Distorted Grid for the sedimentation prediction of EVs, a correlation between the isolation method and the biological and functional effects of EVs was demonstrated [34]. Such findings and the panel of proposed methodologies will be pivotal for the development of optimal isolation methods and to establish EVs as mainstream therapeutics and diagnostics, with significant impact in a wide range of sectors including biopharma and biotechnology, as well as for regulatory agencies.

## 4. Advances in EV Characterization and Biomarker Discovery

During the virtual ISEV2021 Annual Meeting, several oral presentations and posters were focused on innovation and advances in the physico-chemical characterization of EVs for the identification of new biomarkers for diagnostic, prognostic and monitoring purposes, including therapeutic outcome evaluation. Indeed, the field of EV research still faces limitations in the identification of single molecules loaded inside the core of vesicles or exposed on their surface, and would significantly benefit from new achievements in the technological landscape.

To accomplish the goal of single molecule detection, sensitivity and the lowering of the detection limit are essential features of a successful platform. At ISEV2021, multiple biosensors were proposed to ameliorate the detection of EVs in complex biofluids. The need for more and more sensitive sensors to detect EV surface markers also inspired the work by Dr. Sahu and colleagues (Uppsala University, Uppsala, Sweden) who developed an electrokinetic sensor that could be used for highly sensitive EVs’ surface protein profiling. Indeed, sensitivity is one of the most important criteria irrespective of sensing modalities. In the case of surface-based sensors utilizing electrical/electrostatic effects for signal transduction, several attempts to enhance their sensitivity have been previously proposed, and in this case, the sensor surface was coated with positively charged biotinylated copolymers of poly-L-lysine and polyethylene glycol. Streptavidin and avidin were used as linkers, followed by the immobilization of biotinylated antibodies or affinity capture probes against CD9, CD63 and EGFR. The objective was to optimize the experimental setup in order to reach the sensitivity required for the successful application in tumor detection by liquid biopsies still using a limited sample volume (PS05.03) [30] (Figure 2b). Similarly, another electrokinetic sensor was proposed by Dr. Cavallaro (KTH Royal Institute of Technology, Stockholm, Sweden), who described a multiplexed platform to detect and compare tumor markers such as EGFR and PD-L1 in small EVs from pleural effusions of non-small-cell lung cancer patients, demonstrating that the platform may be used for the monitoring of EVs’ alterations during treatments (PS 05.13) [35].

To avoid labelling issues, label-free nanotechnology-based biosensors are also finding increasing application. Microarrays of antibodies were proposed, in combination with the Surface Plasmon Resonance imaging (SPRi) technique, to detect and characterize EVs from human and murine biofluids as biomarkers of treatment effectiveness. In particular, Dr. Gualerzi (IRCCS Fondazione Don Carlo Gnocchi ONLUS, Milan, Italy) presented the potentiality of SPRi to guarantee a multiplexed and sensitive analysis of EVs isolated from liquid biopsies for the discovery of stroke-related biomarkers. The simultaneous detection of different subpopulations of EVs allowed for the evaluation of the relative amount of specific cell-derived EV populations and differences in their cargo during disease progression and rehabilitation-induced recovery (OD21.02). The same methodology was already reported for brain EV characterization [31] (Figure 2c), and was adopted by Dr. Picciolini (IRCCS Fondazione Don Carlo Gnocchi ONLUS, Milan, Italy) for the evaluation of the efficacy of therapeutics, allowing the simultaneous relative quantification of specific neural EVs populations after the administration of a new potent, selective, orally available and brain-penetrable drug that could potentially be used to face a neuroinflammatory insult (CC2.5). The SPR technology was used also by the group of Dr. Im who previously developed a nanoplasmonic sensing platform, termed the nanoplasmonic exosome (nPLEX) [36]. Such a sensor can rapidly and sensitively detect tumor-derived EVs directly from clinical samples, while still presenting some limitations related to bulk analyses, the lysis step requirement and the lack of multiplexed analysis in single EVs. For these reasons, further improvements were proposed and the periodic gold nanohole array was adopted to capture and label EVs for multiplexed EV molecular profiling (PS06.07). A nanoplasmonic platform based on plasmon-enhanced fluorescence detection for single EV analysis was proposed, where the signal, coming from antibodies, that was specific for EV-associated tetraspanins and for cancer EVs was enhanced by the gold nanohole substrate, providing a better understanding of the molecular EV heterogeneity and improving the robustness and accuracy of EV-based cancer detection [37]. Similarly, the University of Coimbra (Biomark@UC, Coimbra, Portugal) presented a biosensor developed to monitor intact EVs circulating in plasma coming from neuronal cells. A biomimetic polymer obtained through molecular imprinting was used to evaluate, with high sensitivity and selectivity, the presence of the external protein glutamate ionotropic receptor AMPA type Subunit 3 (GRIA3), which is specific for neuronal EVs (OD21.04). A new technology for the characterization of single EVs was presented by Dr. Dhande (NanoView Biosciences, Brighton, MA, USA). It is a multiplexed ExoView Flex technology that allows the design of a single EV custom assay that is able to capture EVs with high yield and reproducibility and it could be aligned with other ExoView technologies such as counting, sizing or purification-free single-particle detection (PS05.14). In an interesting work, Dr. Skovronova (University of Turin, Turin, Italy) characterized different fractions of mesenchymal stem cell (MSC)-derived EVs, from different sources, trough different techniques including ExoView to detect size and marker expression, NanoSight to analyze the size and concentration, NanoImager to detect single vesicles and related markers and MACSPlex to assess EV surface markers. This detailed characterization of the EVs required for the safe and effective application of stem cells in clinical applications revealed overlapping results using different instruments (PS05.06).

Recently reviews considered how innovative technologies such as vibrational spectroscopies, spectrometry and other biophotonics-based techniques [19,24] are finding valuable application in the biomedical field; nevertheless, the volumes and amounts of material might be limitations for the application of traditional biophotonics techniques to natural nanoparticles. For example, to overcome such limitations, crosslinking mass spectrometry has been proposed when working with EVs. The strategy proposed by Dr. Bauzá-Martinez and colleagues (Utrecht University, Utrecht, Netherlands) used a highly efficient crosslinker (disuccinimidyl suberate) and a fractionation strategy to enrich for crosslinked peptides. The authors reported the feasibility of using crosslinking mass spectrometry to study the surface interactome of intact B-cell-derived EVs, allowing the simultaneous mapping of several membrane- and EV-specific protein interactions, demonstrating that it could be also used to make structural models to investigate membrane fusion and uptake processes in recipient cells (CC3.1). As recently described by the same authors [38], the crosslinking of peptides to specific surface receptors (i.e., HLA protein complex) makes it possible to best preserve extracellular protein–protein interactions. With such a strategy, no structural artifacts would be introduced, providing native structural information in a manner similar to cryo-electron microscopy of intact cells, without protein tagging or overexpression. On the contrary, Interferometric Light Microscopy was presented by Dr. Berger (Myriade lab, Paris, France) for quick and reliable EV quantification, and was shown to be compatible with the use of multiple larger patient cohorts and with limited volumes of liquid biopsies (Videodrop analysis; PS05.08).

Considering the physico-chemical characterization of EVs and the need for a fast procedure to assess the purity of the EV isolation step, Dr. Ridolfi (University of Florence, Florence, Italy) provided an interesting talk on the study of both the nanomechanical and morphological aspects of EVs by Atomic Force Microscopy (AFM), measuring the contact angle displayed by each vesicle, upon adsorption on a surface (CC3.4). The proposed new application has the advantages of being considerably fast and easy to perform, with limited instrumental requirements. Besides, the analysis demonstrated the ability not only to assess the presence of EVs, but also to detect contaminants within an EV sample and to give a mechanical characterization of multiple EVs coming from distinct natural sources [32].

Thanks to the benefits obtained from HPLC-based studies for the isolation and quantification of EVs, a novel method to improve chromatographic performance during EV isolation was adopted by Dr. Billotto (Clemson University, Clemson, SC, USA). It consists of a trilobal polyester (PETY)C-CP fiber phase employed in a hydrophobic interaction chromatography (HIC) protocol using a Dionex Ultimate 3000 HPLC system that provides greater packing homogeneity than the eight-channel form, and fast isolation and quantification of EVs using low sample volume (PS10.01).

As highlighted by Dr. Lucien (Mayo Clinic, Rochester, MN, USA) during his presentation (OD08.05), some of the advanced technologies proposed in the field of EV research still face some crucial issues, such as reproducibility limits, lack of standardization and inadequate limits of detection for the discrimination of EVs from artifacts such as reagent aggregates. Such concerns often slow the translation to clinics of EV research and limit the undertaking of multicentered studies for the validation of results. In this framework, considering the growing application of nanoscale flow cytometry (nFC) in biomedical research, in particular in diagnostics [19], the importance of calibration and standardization was stressed for all characterization methods. Indeed, nFC is a powerful and rapid method that combines fluorescence and light-scattering detection and the spreading of its application requires the identification of the optimal settings and recommendations for the reproducible enumeration of EVs. Recent observations were presented regarding the use of nFC for the analysis of EVs in biofluids such as the blood and urine of healthy donors and prostate cancer patients, testing 12 different acquisition settings, suggesting critical factors for fluorescence detection of EVs, such as, for example, the number of dyes per antibody and the choice of fluorophores.

Microfluidic geometry is another approach that was optimized for the efficient capture of EVs. Dr. Hisey (University of Auckland, Auckland, New Zealand) developed a chip testing both pillar and herringbone microfluidic geometries and optimizing parameters such as periodic unit cell approximation, flow rate and channel height, using supercomputers. This work demonstrated the potential of simulations of microfluidic EV liquid biopsy in advancing these devices towards clinical applications (PS10.12).

In the search for a reliable, easy and specific method to distinguish cancer cell-derived EVs, nucleic-acid aptamers have been proposed as a promising class of molecules that serve as high-affinity ligands of disease-associated proteins, despite possible non-specific binding risks. For example, the SELEX strategy was developed and validated to isolate aptamers that are specific for EVs [33]. The successful application of the exo-SELEX platform allowed the optimization of a high-affinity aptamer for breast cancer-derived EVs to be used as both an early diagnostic and a prognostic biomarker, but also as a therapeutic target. Indeed, it was found that the selected sequence (aptamer ex-50.T) inhibited the exosome’s cellular uptake, thus antagonizing cancer exosome-induced cell migration (OD14.07) [33]. Specific aptamers were also used for the functionalization of a platform based on metal organic framework, proposed and tested to isolate EVs from breast cancer cell lines with a specific membrane protein. Combined with the rapid nucleic acid isothermal detection method, the difference in the number of EV subpopulations can be converted into the difference in fluorescence signal intensity. This integrated method can be potentially used for rapid detection of specific EV families (PS05.11).

Finally, a paper-based rapid diagnostic test based on the use of DNA probes and gold nanoparticles as colorimetric labels to detect EV-associated miRNAs was proposed. Dr. Rubio-Monterde (Paperdrop Dx, Barcelona, Spain) tested this assay for the detection of miRNA-638 loaded in serum EVs, as it is associated with atherosclerotic plaque vulnerability and the risk of suffering an ischemic stroke (PS16.13). A limit of detection of 10 ng/mL was calculated, demonstrating also the capability to perform a semi quantitative analysis for the assessment of ischemic stroke risk in a rapid, user-friendly and affordable way.

## 5. Advances in EV Application for Drug Delivery

Thanks to the nanoscale dimensions of EVs and their ability to transport bioactive molecules, EVs are widely studied as valuable vectors for drug delivery. This field of research takes advantage of the biocompatibility of vesicles and their targeting. Still, modifications have been proposed to increase their efficiency and specific targeting, including genetic engineering of the parental cells, lipid insertion, affinity interactions and chemical conjugation. At ISEV2021, Jayasinghe (National University of Singapore, Singapore) described a novel approach for the surface functionalization of EVs based on enzymatic ligation, enabling the efficient and specific delivery of encapsulated drugs, such as chemotherapeutics, peptides and RNA, to target cells (CC3.2). The method involves the use of protein ligases to conjugate molecules onto EV membrane proteins via the formation of covalent peptide bonds, preserving biocompatibility and improving the efficiency and versatility of existing methods [39].

The beneficial effects of MSC-derived EVs in regenerative medicine, wound healing and immunomodulation have been widely demonstrated; still, problems remain regarding the standardization and scaling up of their production, their quality control and the possibility of prolonging their half-life. Considering the rapid clearance of EVs from the body, a gelatin methacrylate hydrogel, using 3D-printing, was proposed by Dr. Born from the University of Maryland (College Park, MD, USA). The hydrogel allows for a release of EVs within a 24 h period showing retained bioactivity in HUVEC cells. The printing method has advantages for the tailoring of release profiles for optimized wound healing (PS 09.01). In another work, Dr. Novoseletskaya (Moscow State University, Moscow, Russia) explored the effects of matrix-bound EVs within the native extracellular matrix on the behavior of stem cells, finding that extracellular matrix of human adipose-derived MSC includes matrix-bound vesicles that regulate the functional activity of stem cells that interact with the matrix (PS08.15). Moreover, to measure some modulatory effects of MSC-derived EVs on the innate and acquired immune system, the feasibility of a macrophage and a lymphocyte cell line was evaluated through the combination of two simple in vitro functional assays. Dr. De Lazzari (University of Padova, Padua, Italy) showed the precision and robustness of this approach, which needs to be further evaluated on a large scale (PS01.11). In the attempt to find an effective strategy for the scalable production of MSC-derived EVs, a novel production strategy was developed based on the Vertical-Wheel bioreactor. This platform allowed a substantial improvement in the production of EVs from MSC that were then validated for their ability to stimulate angiogenesis in a 3D in vitro assay (PS 09.07) [40].

On the other hand, to take advantage of EV structure and biocompatibility, an innovative process was proposed to allow the production of cell-derived vesicles to be used as drug carriers. This innovative technology, called BioDrone, is based on extrusion technology that enables the mass production of EVs, and demonstrates a consistent quality in terms of the conservation of surface markers and engineered components (PS 02.10).

## 6. Conclusions

The sparkling multidisciplinary framework of EV research is finding more and more applications thanks to the implementation of basic biology with innovative technologies. The most recent advances presented at ISEV2021, and reported here, mirror the active landscape of technological integration and cellular biology in the application of EVs for drug delivery and diagnostics.

We strongly believe that further achievements and effective translation to clinics will be significantly accelerated once the two souls of EV research will be fused, i.e., when cell biologists and clinicians will start talking the same language as chemists, engineers and physicists. This requires efforts from both sides, but bases have already been laid to foster the application of EV research findings to solve current unmet clinical needs.

To sum up, the keywords of the ISEV2021 “take-home message” are: (i) *rigor*, in both performing and reporting experiments to guarantee the reproducibility of results; (ii) *accelerate and simplify*—procedures cannot be too laborious, time consuming and expensive to reach the clinical setting; (iii) *scale*—every methodology proposed for both diagnostics and drug delivery should foresee the path to scale up; (iv) *involve*—multicentered studies and multidisciplinary team are the key for successful translation to clinics.
